# Using the CFIR 2.0 Framework to Assess the Implementation of GARDE: A Population Health Management Tool for Hereditary Cancer Risk

**DOI:** 10.21203/rs.3.rs-10070047/v1

**Published:** 2026-07-30

**Authors:** Emily C. Lisi, Guilherme Del Fiol, Anne C. Madeo, Ingrid Wagner, Kimberly A. Kaphingst, Richard L. Bradshaw, Emerson Borsato, Kensaku Kawamoto, Ravi Sharaf, Melissa Frey, Chelsey Schlechter, Caitlin G. Allen

**Affiliations:** Wake Forest University School of Medicine; University of Utah; University of Utah; Wake Forest University School of Medicine; University of Utah; University of Utah; University of Utah; University of Utah; Weill Cornell Medicine; Weill Cornell Medicine; University of Utah; Wake Forest University School of Medicine

## Abstract

**Background:**

Identifying individuals at increased hereditary cancer risk remains challenging in primary care settings due to limited time with providers, low self-efficacy in family history collection, and evolving referral guidelines. Genetic Cancer Risk Detector (GARDE), a population-level open-source software platform, uses electronic health record (EHR)-based family history data and automated patient outreach tools to identify and engage individuals who meet criteria for genetic testing. To better understand how such tools can be integrated into diverse health systems, this study applied the Consolidated Framework for Implementation Research (CFIR) to characterize facilitators, barriers, and key processes involved in implementing GARDE at two large academic medical centers.

**Methods:**

Between September 2023 and June 2025, a total of 36 biweekly meetings with principal investigators and key stakeholders involved in GARDE implementations were recorded, date-stamped, transcribed, verified, and coded using CFIR 2.0 domains and subdomains. Facilitators, barriers, and implementation processes were categorized by each site (n =2) and meeting date. Coding captured site-specific contextual factors, meeting-level variation, and co-occurring themes across the implementation timeline. A secondary coder reviewed the transcripts to further contextualize themes and ensure accuracy in coding.

**Results:**

Principal investigators and stakeholders discussed 22 facilitators and 32 barriers to implementation in 14 of 36 total meetings. Facilitators predominantly reflected individual characteristics, including stakeholder buy-in and leadership support while the barriers most frequently reflected inner setting constraints (e.g., limited IT or staffing resources), implementation climate challenges (e.g., competing institutional priorities), and innovation characteristics (e.g., installation or integration difficulties), which often co-occurred. Implementation processes were similar across the two sites, with each site engaging in teaming, planning, tailoring strategies, and adapting workflows. Key cross-cutting themes included data privacy considerations, patient outreach strategies (e.g., messaging modality and frequency), and differing institutional motivations for implementation.

**Conclusion:**

Findings highlight the importance of coordinated planning, stakeholder engagement, and attention to organizational context when implementing GARDE. These insights will support the development of an implementation toolkit and guide future efforts to integrate population-level genomic decision support tools into routine care.

## Introduction

The importance of risk-stratification in identifying individuals at higher risk of developing cancer has become increasingly evident in the age of precision medicine. For higher risk individuals, more frequent screening starting at a younger age can detect cancer at earlier and more treatable stages.^1–4^ Individuals found to harbor a pathogenic variant in a gene associated with predisposition to cancer are at significantly increased risk, with type of cancer and risk level dependent on which gene and variant are identified. Because of the benefits of early screening and risk-reduction strategies (e.g., chemoprevention, risk-reducing surgery) in these individuals, the U.S. Preventive Services Task Force recommends that individuals with a family or personal history of breast cancer be assessed by their primary care physician (PCP) for risk, and those who meet the guidelines for higher risk be referred for genetic counseling and/or genetic testing.^5^ Similarly, the National Comprehensive Cancer Network (NCCN) recommends that all people with a strong history of breast cancer or pancreatic cancer receive prompted enhanced screenings, respectively.^3^

Unfortunately, PCPs are overburdened and understaffed, with reported low self-efficacy and insufficient time for effective collection of their patients’ family histories.^6–10^ Additionally, the complexity and dynamic nature of recommendations in the guidelines such as those developed by the NCCN contribute to low rates of referrals to genetic counseling.^11^ Using clinical decision support (CDS) tools to automatically review electronic health record (EHR) data and perform risk stratification at the population level can quickly identify individuals who may qualify for genetic testing based on family history data.^12^ Additionally, providing automated outreach, education, and access to genetic testing to these individuals can reduce healthcare provider burden while increasing patients’ access to guideline-recommended healthcare.

One such innovation, the GARDE platform, is an EHR-agnostic population health platform that screens cancer family history information in the EHR to identify individuals who meet criteria for genetic testing for hereditary breast, ovarian, prostate, pancreatic, and/or colorectal cancers.^13,14^ Once identified, GARDE provides an authoring tool (GARDE-Chat) to create chatbots that provide outreach, education, and access to genetic testing,^15^ which has been demonstrated in clinical trials to be equivalent to the standard of care in the uptake of pre-test education and genetic testing.^16,17^ Through research funded by the Informatics Technology for Cancer Research (ITCR) Program of the National Cancer Institute (NCI), GARDE has been implemented in several health care systems throughout the United States. Successful implementation of this type of innovation is challenging given the complexity of the tool and the need for buy-in by multiple stakeholders across the institution.

The objective of this study was to use the Consolidated Framework for Implementation Research (CFIR) 2.0 framework to follow the process of implementing GARDE at two large academic medical center sites by reviewing the recurring progress meetings among the implementation stakeholders.^18,19^ Identifying pertinent facilitators and barriers to successful implementation will enhance the development of best practices for sites employing GARDE or similar innovations in the future. Results will inform the creation of an implementation toolkit for GARDE for these future sites.

## Methods

To facilitate the implementation process, biweekly virtual meetings were held between September 2023 and June 2025 with principal investigators (PIs) from each of the two implementation sites and key implementation stakeholders from sites that had previously implemented or were actively implementing GARDE to discuss successes and pain points. The study was conducted across two large academic healthcare systems in the U.S. Implementation stakeholders from each site included clinical leaders, information technology (IT) directors, genetic counselors, and primary care providers. The discussions at these meetings were recorded and transcribed using the native transcription feature in Microsoft Teams. A member of the author team verified each transcript’s accuracy. Transcriptions were entered into a REDCap database and were then reviewed and coded by a single coder (ACM), using the five domains of the CFIR 2.0 framework (Innovation Characteristics, Outer Setting, Inner Setting/Implementation Climate, Individual Characteristics, and Processes).^18,19^ Instances in which PIs and stakeholders mentioned facilitators or barriers to implementation of GARDE were categorized into the CFIR domain/subdomain, including which site discussed each instance and the date of the discussion. A second coder (ECL) reviewed the transcripts to further analyze the topics being discussed when each of the facilitators and barriers were coded. The number of instances of facilitators and barriers for each of the domains was calculated, and a timeline was developed to analyze at which implementation process of GARDE each domain was relevant. Lastly, domains that were concurrently coded on a meeting date were analyzed for relevance and recurring themes.

## Results

A total of 36 PI and stakeholder group meetings were recorded and transcribed between 9/20/2023 and 6/2/2025. Between 4 and 23 individuals were present at the meetings (mean of 13.3). In 14 of the 36 meetings, a facilitator, a barrier or both was mentioned by one of the implementing sites. The remaining meetings focused on other aspects of grant-related activities. In total, more barriers (N = 32) than facilitators (N = 22) were identified.

## Barriers to Implementation

The CFIR 2.0 domains in which barriers were mentioned included *innovation characteristics* (N = 8, perceptions about GARDE characteristics); *outer setting* (N = 1, economic, political, and social context external to the implementing organization); inner setting (N = 9, structural, political, and cultural contexts within the implementing organization); *implementation climate* (N = 10, cultural and political contexts within the implementation environment); and *individual characteristics* (N = 4, individual mindsets, norms, and interests of the implementation team members) (Table 1).

Implementation climate barriers were most commonly related to competing institutional priorities and limited time and resource availability. Inner setting barriers centered on infrastructure constraints, particularly limited IT capacity and staffing. Innovation characteristics reflected the perceived complexity of GARDE and challenges with system integration and installation. Outer setting barriers were mentioned only once relating to regulatory requirements (e.g., Health Information Portability and Accountability Act of 1996 (HIPAA) compliance) and local sociocultural considerations. Individual level barriers included limited leadership support and challenges with coordination among stakeholders.

Across domains, barriers were not isolated; rather, they frequently co-occurred. A key pattern observed was the co-occurrence of innovation, inner setting, and implementation climate, particularly in the context of technical integration. For example, complexities with GARDE that made installation challenging (innovation characteristics) often required IT resources that were not readily available (inner setting), which in turn delayed progress due to competing institutional priorities (implementation climate).

## Facilitators to Implementation

The domains in which facilitators were identified included: three innovation characteristics (n = 3), inner setting (n = 1), implementation climate (n = 4), and individual characteristics (n = 14) (Table 2). No facilitators were identified in the outer setting domain.

Facilitators were primarily driven by stakeholder engagement and perceived value of the innovation. Individual level facilitators included strong leadership support, stakeholder buy-in, and the presence of institutional champions. Implementation climate facilitators reflected alignment with existing workflows and institutional priorities. Innovation-related facilitators included perceived evidence strength and adaptability of GARDE to local contexts. Inner setting facilitators emphasized effective communication and collaboration across teams.

Notably, facilitators were coded and discussed less frequently than barriers and often reflected a high level of engagement among implementation team members, suggesting that successful implementation relied heavily on motivated PIs and stakeholders to overcome structural and technical challenges.

## Alignment Between Barriers and Facilitators

In reviewing the timeline in which domain facilitators and barriers were discussed per meeting, we found that implementation climate was always discussed as a barrier when innovation characteristics were also identified as a barrier. For example, on 1/27/2025, one stakeholder stated:

That’s been a hold up. That was a total surprise to me [because] I went through- and it took me probably half a day to do implementation on my side- to go add the support for the server. Then, to actually not have it work as it did on our test suite was a pretty big surprise. The going back and forth has taken a while…The turn around time is the hardest part [because] between [name of person] and I, I’ll get my release to him, and it’ll take him two or three days to get to it. He’ll go run it, and we’ll have a meeting, and there’ll be a new error…

When a problem occurred with integrating the GARDE software, the institution did not always have the IT personnel available to solve the problem, thus GARDE complexity and institutional priorities were compounding barriers. Additionally, inner setting barriers were also usually discussed in these conversations - the IT infrastructure was not in place to provide the resources needed to create timely solutions. GARDE requires information technology infrastructure (innovation characteristics; inner setting domain), which can stretch the available resources (implementation climate) of the institution.

## Implementation Process Themes

Instances in which site implementation processes were discussed were also recorded (Table 3). Sites 1 and 2 discussed 26 and 23 total processes, respectively. Both sites 1 and 2 discussed the following processes: teaming (N = 6, intentionally working together on interdependent tasks); assessing needs of GARDE deliverers (N = 1, collecting information about priorities and preferences of stakeholders); planning (N = 3, identifying roles and responsibilities and defining goals); tailoring strategies (N = 1, choosing implementation strategies to address barriers); doing (N = 4, implementing in small steps to trial); and adapting (N = 2, modifying for optimal fit into work processes). Processes discussed by Site 1 but not Site 2 included the following: engaging GARDE deliverers (N = 3, attracting and encouraging participation of stakeholders) and reflecting and evaluating (N = 1, collecting and discussing information about the success of implementation). Site processes discussed by Site 2 but not by Site 1 included the following: assessing needs of GARDE recipients (N = 2, collecting information about priorities and preferences of participants) and assessing context (N = 4, collecting information to identify barriers and facilitators to implementation).

When analyzing the transcripts as a whole, common process themes emerged across both sites. In the assessing needs phase, concerns arose around patient privacy regarding data storage options as well as in communications strategies, such as using text messaging to communicate with patients versus internal EHR messaging (e.g. MyChart). In the planning and engaging phases, the number and frequency of messaging with patients were common topics of discussion: whether sending multiple messages was advantageous versus a single message. Regarding engaging with GARDE deliverers, there were multiple conversations around the utility and creation of “marketing” information, as well as an implementation toolkit to provide new sites that were considering or planning GARDE implementation. Planning also involved how to implement GARDE in a clinical setting (versus the grant-funded research setting in which the 2 sites were involved) and how to make that process most operationally efficient. Additionally, a characteristic of GARDE that was considered a facilitator in the planning phase involved conversations around the accuracy of family history data within the EHR; using a chatbot to communicate with a patient directly can clarify family history data, leading to more accurate referral patterns. Within the tailoring strategies and doing phases, similarity between a new implementation site’s EHR and software and data hosting platforms with sites that have previously implemented GARDE facilitated a straightforward build at the new site. Having different software and data hosting platforms created complexity in communication between the platform and GARDE software. Lastly, regarding reflecting and evaluating, conversations revolved around evaluating the required personnel support for successful implementation at all levels.

## Discussion

Using the CFIR 2.0 framework, pertinent facilitators and barriers to the implementation of GARDE at two new academic medical sites were evaluated by coding recurring PI and stakeholder meetings. Barriers were discussed more commonly than facilitators in the meetings. The most common facilitator was individual characteristics (e.g. having a “champion” to promote GARDE). The outer setting was never mentioned as a facilitator. The most common barriers discussed were inner setting (e.g. limited time and resources), implementation climate (e.g. competing priorities), and innovation characteristics (GARDE software complexity), while outer setting (HIPAA) was only mentioned as a barrier once. Interestingly, implementation climate barriers were always discussed with innovation characteristics barriers, and inner setting barriers were usually discussed with the former two domains as well.

Many of our findings from this study are consistent with the literature discussing the implementation of open-source software platforms, also known as CDS tools. Derksen et al. (2025) performed a systematic review of the literature on the implementation of CDS tools in the primary care setting. Common themes identified in their review of 99 articles included external context barriers (comparable to CFIR’s inner setting barriers) such as financial and resource limitations as well as external context enablers (e.g. inner setting facilitators) such as involving a stakeholder “champion”. Technical barriers and enablers (e.g. intervention characteristic barriers and facilitators) identified across studies included lack of integration into the current EHR system and flexibility of the tool for the given EHR platform, respectively.^12^ Specifically, Rubin et al. (2021) cited technical issues due to security restrictions as a barrier to the implementation process of their CDS tool for detection of esophageal gastric cancer, similar to the technical issues experienced at one of the new implementation sites in our study.^20^ Needing to develop a “work-around” for different IT platforms created significant delays in GARDE implementation.

Campbell et al. (2023) developed a CDS tool for the Epic EHR to track the ordering and disclosure of results of genetic testing in the inpatient setting. Similar to the implementation of GARDE, the authors cited barriers to implementation that included the required investment of many hours of work from a multidisciplinary team of stakeholders, including genetic counselors, clinical genetics providers, clinical informatics, and information services as well as IT complexities that required expertise not available at all institutions. However, also like GARDE, implementation was facilitated by stakeholders who were invested in patient care improvement and were willing to be early adopters of a new system.^21^

Protection of patient privacy was an area of concern during the discussions of GARDE implementation, falling under the outer setting CFIR domain. The HIPAA and subsequent Privacy and Security Rules under HIPAA established standards for protecting protected health information of patients.^22^ Concerns about breaches of patient data privacy and confidentiality using patient-facing CDS tools integrated with the EHR have been raised.^23–26^ Recommendations for CDS implementation planning include involving an institution’s privacy officer for review of all workflows that involve protected health information.

One notable difference between typical barriers using EHR platforms for CDS discussed in the literature and our findings is the chronic problem of inaccurate or missing data being incorporated from incomplete or erroneous medical documentation leading to decreased sensitivity and specificity of the CDS tool.^27–29^ However, GARDE was found to be particularly beneficial in that contacting patients directly via chatbot to add missing family history information can greatly increase sensitivity, which was identified as an innovation characteristic facilitator.

Analyzing the CFIR processes at both implementation sites revealed that each site went through similar processes during the study period. For example, each site was interested in determining type (e.g. patient portal versus text) and frequency of messaging to send for participant recruitment. Patient use of EHR portals for managing their healthcare has increased significantly in the past several years.^30,31^ Given their accessibility to patients, clinical trial teams have started using patient portals for participant recruitment. While more research is necessary on the benefits and limitations of using patient portals for recruitment, preliminary data has indicated that patient portals may be more effective and less costly than traditional methods (e.g., phone or mail outreach), particularly in healthy populations.^32–36^ Patients recommend that portal messages are specific and include enough detail for participants to understand study goals.^34,37^ Of note, in a qualitative study by Miller et al. (2023), participants felt that text messages as well reminder messages through a patient portal were acceptable recruitment tools.^37^ Reminder messages have been found to increase participation rates.^38,39^ However, IRBs may view repeated recruitment contact as a potential source of undue influence. Challenges remain when considering inclusive recruitment efforts, as underrepresented patient populations may be less likely to have access and/or digital and health literacy levels to successfully navigate them.^40,41^ In general, more research is needed on use of patient portals for research recruitment, specifically involving the number of messages that can be sent before participants feel pressured.

Lastly, a novel implementation barrier that emerged in this research was the misalignment of institutional goals across sites. For more research-focused sites, the availability of rich data to foster research studies using GARDE was attractive (innovation characteristic facilitator), whereas community health sites were more concerned with the potential to generate clinical revenue (inner setting/implementation climate barrier).

More broadly, these differences reflect how health systems may prioritize the use of patient portals as tools for communicating with patients, whether to support research and evaluation, facilitate clinical care delivery, or engage patients in care. Academic medical centers who traditionally focus more directly on research rely on grant funding, typically through the National Institutes of Health or other federal/philanthropic agencies. Innovative strategies for research such as CDS tools that help move research forward are appealing in these environments. In the current U.S. climate of cost-cutting initiatives in clinical care reimbursement, especially through state and federal programs (e.g. Medicaid and Medicare), more emphasis may be placed on clinical research to generate institutional revenue.^42^ On the other hand, community health centers often lack the infrastructure and administrative support to carry out research effectively and rely on clinical revenue to operate effectively.^43^ Unfortunately, community health centers are more often located in rural settings and provide care for a larger proportion of underrepresented populations than large academic medical centers. These infrastructure and resource constraints may limit adoption of new patient-facing portal-based technologies, with implications for both research participation and downstream health equity.^44^ It is important, therefore, for implementation sites with access to research resources to develop research partnerships and/or clinical pipelines for community health institutions to engage with new innovations.

There are several limitations to this study that should be acknowledged. PI and stakeholder discussions of barriers and facilitators of GARDE implementation at two large academic medical centers were analyzed by the study team; other implementation sites may have different experiences. Individual site representatives were not available for every meeting due to other obligations, time of work, etc. As discussed above, sites that rely more heavily on clinical rather than research funding—including the implementation sites described in this study—may experience different barriers and facilitators to implementation. Lastly, the original transcripts were analyzed for facilitators/barriers by one coder. Others analyzing the same data may have recognized different facilitators/barriers from the same data.

Implementation of population-level open-source software platforms such as GARDE requires coordinated effort across technical, operational, and clinical stakeholders. This CFIR-guided analysis illustrates how multiple contextual factors—ranging from organizational resources to institutional priorities and stakeholder engagement—interact to influence implementation progress at each site. Although the two participating sites differed in structure and motivation, they encountered similar challenges spanning multiple CFIR domains, underscoring the complex interplay between innovation requirements and local contexts. Importantly, many of the factors that shaped implementation were addressable, highlighting opportunities to streamline adoption at future sites.

These insights will inform the development of a GARDE implementation toolkit designed to support early planning, clarify stakeholder roles, and anticipate common workflow and technical considerations. Future work should evaluate the impact of the implementation toolkit, explore GARDE deployment in additional clinical environments—including community and resource-limited settings—and assess long-term outcomes such as scalability, sustainability, and patient reach. As genomic risk assessment becomes increasingly integrated into routine care, ensuring that tools like GARDE can be adopted efficiently across diverse health systems will be essential for promoting equitable access to hereditary cancer evaluation.

## Supplementary Material

This is a list of supplementary files associated with this preprint. Click to download.

• Supplementarymaterial.docx

## Figures and Tables

**Table 1:** GARDE Implementation Barriers Delineated by the CFIR Domains

CFIR Domain	Innovation Characteristics	Implementation Climate	Inner Setting	Individual Characteristics	Outer Setting	Total
**N/(%)**	N = 8/(25.0%)	N = 8/(25.0%)	N = 9/(28.1%)	N = 6/(18.8%)	N = 1/(0.03%)	N = 32/(100.0%)
**Site 1**	1	3	2	2	0	8
**Site 2**	2	2	2	2	0	8
**Both**	5	3	5	2	1	16
Illustrative Quotes	“So, on our end, we just downloaded GARDE on Friday, ran into some installation errors that we’re gonna go through this afternoon to run it.” (1/13/25)	“Certainly, that was kind of what they described-seemed to me sort of a ‘how are we going to justify paying for the human who will need to go through records and things, and you know, they had a lot of very specific questions about things like insurance, capitation, and stuff like that.” (4/1/24)	“The next step after this is going to be chatbot installed. We considered the possibility of having them run in parallel. Unfortunately, locally, we just don’t have the resources to do so.” (1/27/25)	“They’re both working, independently, but they need to talk to each other.” (4/21/25)	“The bottom line I got was there was a lot of discomfort with using text messaging for, in particular, collecting PHI due to the sensitivity and the fact that HIPAA requires that, if you are using text messaging or email to communicate with patients, because they’re considered unsecure channels, that we get explicit approval from patients to do so wherein we notify them of the fact that text messaging, for example, is a unsecure channel and to get their explicit consent to do so.” (1/27/25)	

**Table 2:** GARDE Implementation Facilitators Delineated by the CFIR Domains

CFIR Domain	Innovation Characteristics	Implementation Climate	Inner Setting	Individual Characteristics	Outer Setting	Total
**N/%**	N = 4/18.1%	N = 3/13.6%	N = 1/0.05%	N = 14/64.4%	N = 0/0.0%	N = 22/100.0%
**Site 1**	1	1	1	9		
**Site 2**	1	1	0	3		
**Both**	2	1	0	2		
Illustrative Quotes	“The notion of not trusting the data that’s in the EHR- I think what’s unique about GARDE that could be helpful here, it’s the ability to reach out to patients to get information, validate it, update it, so I think that’s a neat angle where we can get [buy in].” (1/13/25)	“I think those numbers are important and just emphasizing USPSTF, at least in my sample size of places I’ve been, primary care generally will want to make sure USPSTF is supported, so I think that angle is really good for primary care.” (6/24/24)	“We used the Utah Population Database, which has genealogy records back from the 1800s. And it’s integrated with a number of data sources like the vital records with birth and death certificates and also the cancer registry. So it has extensive data about family history using those data sources. So, we tested what would happen if we try to augment family history from the EHR using data from the [an external population database]] and we found that we almost doubled the identification rate of individuals meeting criteria for genetic testing.” (6/10/24)	“Getting a champion who’s institutionally important would be good and references is really important too, so what could be powerful is if there was this group who can be available as references to say like, ‘Hey. This was great. It was easy. We can’t share the data, but it increased our revenue.’ That kinda thing would be quite I think useful.” (4/29/24)	N/A	

**Table 3:** CFIR Processes and Subprocesses Mentioned by Site with Illustrative Quotes

Processes	Subprocesses	Site 1	Site 2	Quote
Teaming	N/A	6	6	“And we’ve been able to collaborate with them in the recruitment of, you know, personnel; also the purchase of a large infrastructure IT infrastructure that includes a data warehouse, includes patient outreach, you know, text messaging campaigns, that kind of thing.” (4/1/24)
Assessing Needs	GARDE Deliverers	4	1	“It’s, if we’re trying to convince some particular health system to purchase, their obvious question is not gonna be: ‘If we spend, you know, $30,000 on GARDE, not how much revenue generally we can make as well, but like how much clinical profitability can we expect for that $30,000?’ You know that kind of thing where they would want to see $30,000 at least. (9/16/24)
	GARDE Recipients	0	2	
Assessing Context	N/A	0	4	“It seems like both sites are very interested in text versus MyChart or different approaches within MyChart or timing and so maybe we could think through some AB testing or some randomization that we could do around the initial outreach across both sites.” (5/13/24)
Planning	N/A	3	3	“Definitely, I would say that there’s gotta be some level of QA. It’s not gonna be a ‘you just went through some mappings and you’re done.’ It definitely takes some oversight and some people that know what they’re looking for to make sure everything’s working as expected.” (4/15/24) One of the challenges–we do need a lot of different kinds of people to do, you know, and sometimes it’s a little task here, a little task there. Is to line up all the resources that are need, all of the human resources, that are needed up front, and, and there’s also a sequence in how they get involved.” (10/28/24) But I think if we can lay the entire project out, initially, I think that’s going to help in planning and maybe we can multi track, multi thread, some of the things instead of taking one thread at a time.” (10/28/24)
Tailoring Strategies	N/A	3	1	It’s the kind of thing with large language models, it’s a tricky territory to navigate because if you don’t mention, people will question you’re outdated. If you say, “Oh, by the way, we support OpenAI tools, then it could raise concerns immediately, all sorts of concerns about privacy with the data going to Microsoft or OpenAI. Will it hallucinate? We have to be careful about how do we thread those waters in terms of trying to find a—and it depends on the audience.” (4/29/24)
Engaging	GARDE Deliverers	4	0	“The goal of it was that it would be something that somebody within a organization who was interested in implementing GARDE might be able to easily share with a healthcare system leader to help them understand the potential benefits of GARDE.” (4/29/24)
	GARDE Recipients	0	0	“So what can be done to maximize people clicking on the link that and one of the activities is uh creating more effective communication.” (5/13/24)
Doing	N/A	3	4	: “In my mind, it’s really getting us through this first use case, which is really feasibility of us being able to run everything from start to finish and make sure that all of that is working in the way that it’s designed to.” (4/15/24)
Reflecting and Evaluating	Implementation	1	0	“Yeah. Yeah, I think to that point, it might be worth us going through the steps, just rewind the tape. Look from the beginning. Look at every single step and think through whether there’s opportunities to automate the process or package into multiple steps into one.10/28/24
Adapting	N/A	3	2	“Another one, this could be multiple or standalone ones for each site demonstrating site specific modifications and GARDE as it was deployed at each site.” (7/8/24)
**Total**		**27**	**23**	

## Data Availability

Transcripts from the coded meetings may be available from the corresponding author upon reasonable request. Selected excerpts are included in the manuscript and supplementary materials.

## Abbreviations

CDS: Clinical Decision Support
CFIR: Consolidated Framework for Implementation Research
EHR: Electronic Health Record
GARDE: Genetic Cancer Risk Detector
HIPAA: Health Insurance Portability and Accountability Act
ITCR: Informatics Technology for Cancer Research
NCCN: National Comprehensive Cancer Network
NCI: National Cancer Institute
PCP: Primary Care Provider
REDCap: Research Electronic Data Capture

## References

[1] Issaka RB, Chan AT, Gupta S. AGA Clinical Practice Update on Risk Stratification for Colorectal Cancer Screening and Post-Polypectomy Surveillance: Expert Review. Gastroenterology. Nov 2023;165(5):1280–1291. doi:10.1053/j.gastro.2023.06.033 37737817 PMC10591903

[2] Michaels E, Worthington RO, Rusiecki J. Breast Cancer: Risk Assessment, Screening, and Primary Prevention. Med Clin North Am. Mar 2023;107(2):271–284. doi:10.1016/j.mcna.2022.10.007 36759097

[3] Rex DK, Boland CR, Dominitz JA, Colorectal cancer screening: Recommendations for physicians and patients from the U.S. Multi-Society Task Force on Colorectal Cancer. Gastrointest Endosc. Jul 2017;86(1):18–33. doi:10.1016/j.gie.2017.04.003 28600070

[4] Wong MC, Wong SH, Ng SC, Wu JC, Chan FK, Sung JJ. Targeted screening for colorectal cancer in high-risk individuals. Best Pract Res Clin Gastroenterol. Dec 2015;29(6):941–51. doi:10.1016/j.bpg.2015.09.006 26651255

[5] U. S. Preventive Services Task Force, Nicholson WK, Silverstein M, Screening for Breast Cancer: US Preventive Services Task Force Recommendation Statement. JAMA. Jun 11 2024;331(22):1918–1930. doi:10.1001/jama.2024.5534 38687503

[6] Acheson LS, Wiesner GL, Zyzanski SJ, Goodwin MA, Stange KC. Family history-taking in community family practice: implications for genetic screening. Genetics in medicine : official journal of the American College of Medical Genetics. May–Jun 2000;2(3):180–5. doi:10.1097/00125817-200005000-00004 11256663

[7] Gramling R, Nash J, Siren K, Eaton C, Culpepper L. Family physician self-efficacy with screening for inherited cancer risk. Ann Fam Med. Mar–Apr 2004;2(2):130–2. doi:10.1370/afm.60 15083852 PMC1466652

[8] Rich EC, Burke W, Heaton CJ, Reconsidering the family history in primary care. J Gen Intern Med. Mar 2004;19(3):273–80. 10.1111/j.1525-1497.2004.30401.x PMC1492151 15009784

[9] Taber P, Ghani P, Schiffman JD, Physicians’ strategies for using family history data: having the data is not the same as using the data. JAMIA Open. Oct 2020;3(3):378–385. doi:10.1093/jamiaopen/ooaa035 34632321 PMC7660959

[10] Ahmed S, Hayward J, Ahmed M. Primary care professionals’ perceptions of using a short family history questionnaire. Family practice. Dec 2016;33(6):704–708. doi:10.1093/fampra/cmw080 27535332

[11] Hampel H, Sweet K, Westman JA, Offit K, Eng C. Referral for cancer genetics consultation: a review and compilation of risk assessment criteria. Journal of medical genetics. Feb 2004;41(2):81–91. doi:10.1136/jmg.2003.010918 14757853 PMC1735676

[12] Derksen C, Walter FM, Akbar AB, The implementation challenge of computerised clinical decision support systems for the detection of disease in primary care: systematic review and recommendations. Implementation science : IS. Jul 17 2025;20(1):33. doi:10.1186/s13012-025-01445-4 40671071 PMC12269258

[13] Bradshaw RL, Kawamoto K, Kaphingst KA, GARDE: a standards-based clinical decision support platform for identifying population health management cohorts. Journal of the American Medical Informatics Association : JAMIA. Apr 13 2022;29(5):928–936. doi:10.1093/jamia/ocac028 35224632 PMC9006693

[14] Mowery DL, Kawamoto K, Bradshaw R, Determining Onset for Familial Breast and Colorectal Cancer from Family History Comments in the Electronic Health Record. AMIA Jt Summits Transl Sci Proc. 2019;2019:173–181. 31258969 PMC6568127

[15] Del Fiol G, Borsato E, Bradshaw RL, GARDE-Chat: a scalable, open-source platform for building and deploying health chatbots. Journal of the American Medical Informatics Association : JAMIA. Mar 1 2026;33(3):593–602. doi:10.1093/jamia/ocaf211 41524720 PMC12798686

[16] Kaphingst KA, Kohlmann WK, Lorenz Chambers R, Uptake of Cancer Genetic Services for Chatbot vs Standard-of-Care Delivery Models. JAMA Network Open. 2024;7(9)doi:10.1001/jamanetworkopen.2024.32143 PMC11385050 39250153

[17] Harris A, Bather JR, Kawamoto K, Determinants of Breast Cancer Screening Adherence During the COVID-19 Pandemic in a Cohort at Increased Inherited Cancer Risk in the United States. Cancer control : journal of the Moffitt Cancer Center. Jan–Dec 2024;31:10732748241272727. doi:10.1177/10732748241272727 PMC11489983 39420801

[18] Damschroder LJ, Aron DC, Keith RE, Kirsh SR, Alexander JA, Lowery JC. Fostering implementation of health services research findings into practice: a consolidated framework for advancing implementation science. Implementation science : IS. Aug 07 2009;4:50. doi:10.1186/1748-5908-4-50 19664226 PMC2736161

[19] Damschroder LJ, Reardon CM, Widerquist MAO, Lowery J. The updated Consolidated Framework for Implementation Research based on user feedback. Implementation science : IS. Oct 29 2022;17(1):75. doi:10.1186/s13012-022-01245-0 36309746 PMC9617234

[20] Rubin G, Walter FM, Emery J, Electronic clinical decision support tool for assessing stomach symptoms in primary care (ECASS): a feasibility study. BMJ open. Mar 18 2021;11(3):e041795. doi:10.1136/bmjopen-2020-041795 PMC7978254 33737422

[21] Campbell IM, Karavite DJ, McManus ML, Clinical decision support with a comprehensive in-EHR patient tracking system improves genetic testing follow up. Journal of the American Medical Informatics Association : JAMIA. Jun 20 2023;30(7):1274–1283. doi:10.1093/jamia/ocad070 37080563 PMC10280356

[22] U.S. Department of Health and Human Services. HIPAA Security Rule To Strengthen the Cybersecurity of Electronic Protected Health Information. 2026. https://www.federalregister.gov/documents/2025/01/06/2024-30983/hipaa-security-rule-to-strengthen-the-cybersecurity-of-electronic-protected-health-information

[23] Goldberg DG, Soylu TG, Hoffman CF, Kishton RE, Cronholm PF. Clinicians’ perspectives on the adoption and implementation of EMR-integrated clinical decision support tools in primary care. Digit Health. Jan–Dec 2025;11:20552076251334043. doi:10.1177/20552076251334043 PMC12035066 40297364

[24] Carini C, Seyhan AA. Tribulations and future opportunities for artificial intelligence in precision medicine. J Transl Med. Apr 30 2024;22(1):411. doi:10.1186/s12967-024-05067-0 38702711 PMC11069149

[25] El Arab RA, Al Moosa OA, Sagbakken M, Integrative review of artificial intelligence applications in nursing: education, clinical practice, workload management, and professional perceptions. Frontiers in public health. 2025;13:1619378. doi:10.3389/fpubh.2025.1619378 40823249 PMC12354398

[26] He W, Chima S, Emery J, Perceptions of primary care patients on the use of electronic clinical decision support tools to facilitate health care: A systematic review. Patient Educ Couns. Aug 2024;125:108290. doi:10.1016/j.pec.2024.108290 38714007

[27] Chishtie J, Sapiro N, Wiebe N, Use of Epic Electronic Health Record System for Health Care Research: Scoping Review. J Med Internet Res. Dec 15 2023;25:e51003. doi:10.2196/51003 38100185 PMC10757236

[28] Teno JM. Garbage in, Garbage out-Words of Caution on Big Data and Machine Learning in Medical Practice. JAMA Health Forum. Feb 3 2023;4(2):e230397. doi:10.1001/jamahealthforum.2023.0397 36795395

[29] Johnson D, Del Fiol G, Kawamoto K, Genetically guided precision medicine clinical decision support tools: a systematic review. Journal of the American Medical Informatics Association : JAMIA. Apr 19 2024;31(5):1183–1194. doi:10.1093/jamia/ocae033 38558013 PMC11031215

[30] Johnson C, Richwine C, Patel V. Individuals’ Access and Use of Patient Portals and Smartphone Health Apps, 2020. ASTP Health IT Data Brief. 2021:1–14.

[31] Carini E, Villani L, Pezzullo AM, The Impact of Digital Patient Portals on Health Outcomes, System Efficiency, and Patient Attitudes: Updated Systematic Literature Review. J Med Internet Res. Sep 8 2021;23(9):e26189. doi:10.2196/26189 34494966 PMC8459217

[32] Gleason KT, Ford DE, Gumas D, Development and preliminary evaluation of a patient portal messaging for research recruitment service. J Clin Transl Sci. Feb 2018;2(1):53–56. doi:10.1017/cts.2018.10 31660218 PMC6799557

[33] Ziegenfuss JY, Sour EU, Roelofs EJ, Vesely JM, Margolis KL, Hooker SA. A randomized study comparing patient portal and email communications for trial recruitment. Clinical trials (London, England). Oct 2025;22(5):597–606. doi:10.1177/17407745251358259 40836901 PMC12373006

[34] Plante TB, Gleason KT, Miller HN, Recruitment of trial participants through electronic medical record patient portal messaging: A pilot study. Clinical trials (London, England). Feb 2020;17(1):30–38. doi:10.1177/1740774519873657 31581836 PMC6992491

[35] Sherman SE, Langford AT, Chodosh J, Hampp C, Trachtman H. Use of patient portals to support recruitment into clinical trials and health research studies: results from studies using MyChart at one academic institution. JAMIA Open. Dec 2022;5(4):ooac092. doi:10.1093/jamiaopen/ooac092 36325306 PMC9614350

[36] Samuels MH, Schuff R, Beninato P, Effectiveness and cost of recruiting healthy volunteers for clinical research studies using an electronic patient portal: A randomized study. J Clin Transl Sci. Dec 2017;1(6):366–372. doi:10.1017/cts.2018.5 29707259 PMC5916095

[37] Miller HN, Lindo S, Fish LJ, Describing current use, barriers, and facilitators of patient portal messaging for research recruitment: Perspectives from study teams and patients at one institution. J Clin Transl Sci. 2023;7(1):e96. doi:10.1017/cts.2023.522 37125060 PMC10130833

[38] Koitsalu M, Eklund M, Adolfsson J, Gronberg H, Brandberg Y. Effects of pre-notification, invitation length, questionnaire length and reminder on participation rate: a quasi-randomised controlled trial. BMC medical research methodology. Jan 5 2018;18(1):3. doi:10.1186/s12874-017-0467-5 29304734 PMC5756335

[39] Ley C, Duan H, Parsonnet J. Recruitment into antibody prevalence studies: a randomized trial of postcards vs. letters as invitations. BMC medical research methodology. Jul 22 2023;23(1):170. doi:10.1186/s12874-023-01992-8 37481522 PMC10363298

[40] Tabriz AA, Fleming PJ, Shin Y, Challenges and opportunities using online portals to recruit diverse patients to behavioral trials. Journal of the American Medical Informatics Association : JAMIA. Dec 1 2019;26(12):1637–1644. doi:10.1093/jamia/ocz157 31532482 PMC6857600

[41] Beaton M, Jiang X, Minto E, Using patient portals for large-scale recruitment of individuals underrepresented in biomedical research: an evaluation of engagement patterns throughout the patient portal recruitment process at a single site within the All of Us Research Program. Journal of the American Medical Informatics Association : JAMIA. Oct 1 2024;31(10):2328–2336. doi:10.1093/jamia/ocae135 38917428 PMC11413441

[42] Azoulay P, Heggeness M, Kao J. Medical research and health care finance: Evidence from Academic Medical Centers. Research Policy. 2025;54(2)doi:10.1016/j.respol.2024.105163 PMC13475792 42603013

[43] Likumahuwa S, Song H, Singal R, Building research infrastructure in community health centers: a Community Health Applied Research Network (CHARN) report. Journal of the American Board of Family Medicine : JABFM. Sep–Oct 2013;26(5):579–87. doi:10.3122/jabfm.2013.05.130025 24004710 PMC4559143

[44] Loh IK, Miller TE, Aggarwal R, Richmond TM. Improving Representation of Underserved Populations in Cardiovascular Research. Journal of the American Heart Association. Sep 16 2025;14(18):e038020. doi:10.1161/JAHA.124.038020 40932113 PMC12554393

